# MH-ICP-MS Analysis of the Freshwater and Saltwater Environmental Resources of Upolu Island, Samoa

**DOI:** 10.3390/molecules25214871

**Published:** 2020-10-22

**Authors:** Sasan Rabieh, Odmaa Bayaraa, Emarosa Romeo, Patila Amosa, Khemet Calnek, Youssef Idaghdour, Michael A. Ochsenkühn, Shady A. Amin, Gary Goldstein, Timothy G. Bromage

**Affiliations:** 1 Department of Molecular Pathobiology, New York University College of Dentistry, 345 East 24th Street, New York, NY 10010, USA; khemet.calnek@nyu.edu; 2 Environmental Genomics Lab, Biology Program, Division of Science and Mathematics, New York University Abu Dhabi, Saadiyat Island, P.O. Box 129188, Abu Dhabi 51133, UAE; ob733@nyu.edu (O.B.); youssef.idaghdour@nyu.edu (Y.I.); 3 Hydrology Division, Ministry of Natural Resources and Environment, Level 3, Tui Atua Tupua Tamasese Efi Building (TATTE), Sogi., P.O. Private Bag, Apia 95564, Samoa; emarosa.romeo@mnre.gov.ws; 4 Faculty of Science, National University of Samoa, P.O. Box 1622, Apia 95564, Samoa; p.amosa@nus.edu.ws; 5 Marine Microbial Ecology Lab, Biology Program, New York University Abu Dhabi, Saadiyat Island, P.O. Box 129188, Abu Dhabi 51133, UAE; mao13@nyu.edu (M.A.O.); samin@nyu.edu (S.A.A.); 6 College of Dentistry, New York University, 345 East 24th Street, New York, NY 10010, USA; gary.goldstein@nyu.edu; 7 Department of Biomaterials, New York University College of Dentistry, 345 East 24th Street, New York, NY 10010, USA

**Keywords:** MH-ICP-MS, elemental analysis, freshwater, saltwater, water quality, Upolu Island, Samoa, biosecurity, coral reefs

## Abstract

The elemental composition of freshwater and saltwater samples around the South Pacific island of Upolu, Samoa has been investigated together with other indicators of water quality. Up to 69 elements from Li (3) to U (92) are measured in each sample, analyzed by Mattauch–Herzog-inductively coupled plasma-mass spectrometry (MH-ICP-MS). One hundred and seventy-six samples were collected from surface freshwater sources (24 rivers, two volcanic lakes, one dam) and from seawater sources from the surface to 30 m depth (45 inner reef, reef, and outer reef locations) around Upolu Island, including river mouths and estuaries. Principal component and hierarchical clustering correlation analyses were performed on quantile normalized log transformed elemental composition data to identify groups of samples with similar characteristics and to improve the visualization of the full spectrum of elements. Human activities, such as the use of herbicides and pesticides, may relate to observed elevated concentrations of some elements contained in chemicals known to have deleterious obesogenic effects on humans that may also cause coral reef decline. Furthermore, the salinity of some saltwater samples tested were very high, possibly due to climate variability, which may additionally harm the health and biodiversity of coral reefs.

## 1. Introduction

The quality of freshwater and saltwater resources in relation to their elemental composition, salinity, and many other water quality parameters, such as microbial diversity and microplastics content, is a serious contemporary environmental concern for aquatic life and for humans that depend on these waters. To better understand the potential negative health effects of excesses and deficiencies of element concentrations, an entire elemental analysis is necessary. Apart from biosphere processes, there are numerous human activities that generate anthropogenic effects on top of natural elemental compositions of various water resources with a consequence to their quality for sustaining life [1,2,3,4,5,6,7].

For the determination of the complete inorganic spectrum of elements in aqueous samples, there are several analytical techniques currently in use, such as electrothermal atomic absorption spectroscopy (ETAAS), inductively coupled plasma optical emission spectrometry (ICP-OES), and various types of inductively coupled plasma-mass spectrometry (ICP-MS) [8,9,10,11]. However, these techniques are typically limited in the practical number of elements that can be analyzed from a single sample. Recently, we have developed a method for simultaneous multi-element detection across the breadth of the chemical periodic table in various aqueous samples using a Mattauch–Herzog geometry-inductively coupled plasma-mass spectrometry (MH-ICP-MS) [12].

The Mattauch–Herzog geometry has a configuration of electrostatic and magnetic fields that spreads ions over the complete mass range and distributes them along a flat plane [13]. In contrast to a multicollector-ICP-MS, the MH-ICP-MS uses a single 4800 pixel element detector permitting the simultaneous detection of isotopes over the full relevant inorganic mass spectrum from ^6^Li to ^238^U (SPECTRO MS, SPECTRO Analytical Instruments GmbH, Kleve, Germany). Technical specifications are available elsewhere [14]. Targeted evaluations may be performed on any number of elements desired, or else discovery-based research, otherwise impractical using conventional ICP-MS, may be used across the whole of the mass range.

A principal advantage of this MH geometry is its embracing of the entire inorganic mass range from ^6^Li to ^238^U in a single assay. This helps to ease the limit on the number of inorganic elements whose concentrations may be routinely measured from one sample. It also reduces the operating time and sample volumes for evaluations across the breadth of the periodic table [12].

Driven by knowledge of coral reef and human health decline on Upolu Island, Samoa, our mission is to evaluate the ecological stoichiometry of the Island, which relates to the distributions and concentrations of elements in the environment, and to assess the islands’ biosecurity of water resources in relation to aquatic water quality and human health. This mission is designed to complement the concerns of threats to Upolu Island environments due to global climate change, global warming, and increasing seawater temperatures [15], as well threats from overfishing, sedimentation, microbial and chemical pollutants, and waste disposal [16].

Generally, a very limited number of elements are investigated for environmental water monitoring purposes around Upolu Island. To assess water quality, some of the physical, chemical, and microbiological parameters of Vaisigano River in Upolu Island have been investigated. Lead and copper were considered in this study [17]. An investigation of the chemical composition of Upolu Island drinking water supplies have included analyses of copper, lead, chloride, fluoride, and nitrate anions from Falelatai, Aufaga, and Letogo rivers [18].

We enter into this study knowing that many elements, such as sodium, calcium, and selenium [19,20], are essential to life, but also that many heavy metals have no biological function and are harmful to life in even relatively moderate concentrations. Cadmium (Cd), lead (Pb), and mercury (Hg), for instance, are particularly egregious to life, and their distributions and concentrations are typically a result of industrial and human activity [21,22,23]. However, we also know that excessively high amounts of some essential elements in the body can be toxic. Rare earth elements (REEs) known as lanthanides with scandium and yttrium have no biological function and therefore are considered not to be biologically important [24].

To the best of our knowledge, this is the first comprehensive study performed in the freshwater and saltwater resources of Upolu Island. This study was conducted under the patronage of Honorable Tuilaepa Dr. Sailele Malielegaoi, Prime Minister of Samoa. The Government of Samoa with the assistance of the United Nations Development Programme will use these findings as part of Samoa’s next report to the United Nations Convention on Biological Diversity.

We analyze up to 69 elements in each sample simultaneously in a single run using MH-ICP-MS. There are several objectives of this study. We analyze the elemental composition across the breadth of the chemical periodic table from various freshwater, saltwater, and water samples from transitional locations such as mangrove swamps and estuaries around Upolu Island using our recently developed MH-ICP-MS method. We also provide a basic water quality assessment. We then investigate the possible relationship between the aforementioned analyses with the current status of aquatic and human health. Finally, we provide recommendations that local authorities may use to help mitigate the negative impacts of anthropogenic activities and to restore healthy environmental conditions.

## 2. Results

### 2.1. Multi-Element Determination in Freshwater Samples

The results of multi-element analysis including the range and mean values of 67 freshwater (FW) samples using a MH-ICP-MS are summarized in Table 1. Among major and minor elements, chlorine, sodium, calcium, silicon, and magnesium were dominant in freshwater samples with the concentration range (µg/L) of Cl: 8.9–133,023; Na: 1277–90,863; Ca: 915–24,663; Si: 1564–15,887; and Mg: 893–19,571. All these elements but Cl were present in all investigated samples, and chlorine was not detected in 14 samples. The highest concentration of chlorine and sodium were obtained in FW 09 (Faleaseela River). FW 61 (Vailima River), FW 07 (Faleaseela River), and MW 03 (Mulivaifagatola River) have the highest concentrations of calcium, silicon, and magnesium, respectively.

Lithium was only detected in FW 09 sample (Faleaseela River) with the concentration of 1.46 µg/L. Five elements, namely beryllium (Be), indium (In), osmium (Os), gold (Au), and mercury (Hg), were below the detection limit.

Of all samples, the full spectrum of rare earth elements (REEs) were detected in two samples, FW 55 and FW 56 in the Tiavea River. Most REEs’ concentrations were below detection limits in other analyzed samples. However, the concentration of those elements detected in some of the samples was not higher than 0.20 µg/L.

The highest content of bromine (Br) was detected in two samples, FW 09 (Faleaseela River) and MW 03 (Mulivaifagatola River), with the concentration of 853 and 378 µg/L, respectively. The highest content of strontium (Sr) was obtained in MW 03 (Mulivaifagatola River) and FW 09 (Faleaseela River) samples with the concentrations of 252 and 109 µg/L, respectively.

Most of so-called Mix 2 elements (Sb, Ge, Au, Hf, Ir, Mo, Nb, Pd, Pt, Re, Rh, Ru, Ta, Sn, Ti, W, and Zr) were either not detected or were at very low µg/L or at ng/L range, except the titanium (Ti), which was detected at the concentration of about 100 µg/L in two samples of FW 55 and FW 56 (from Tiavea River).

While Iron (Fe) was not detected or was at low µg/L range, the higher amounts of 848, 775, 585, 395, and 355 µg/L were detected for the five samples of FW 55, FW 56, and FW 40 (Mulivaifagatola River), FW 50 (Taelefaga River), and FW 64 (Vaisigano River), respectively.

The concentration of trace elements of manganese (Mn), cobalt (Co), nickel (Ni), and copper (Cu) were either not detected or were at a very low µg/L or at ng/L range, except for samples FW 55 and FW 56 (both from Tiavea River), which were relatively the same and at about 6, 0.3, 5, and 1.5 µg/L, respectively.

Because the range of concentrations was large, a log transformation of the data was not sufficient to visualize all element concentration variability, particularly among the freshwater sources. For this reason, to log transformed data we applied quantile normalization for the samples to generate a common unit for all element concentrations. Quantile normalization is based on density-adjusted rank ordering making distributions identical in statistical properties and spreading the resultant out between the values 0 and 1, i.e., from no concentration detected to the maximum concentration respectively. This reveals structure in the visualization of the data (Supplementary Figure S1) and allows comparisons of element across samples.

### 2.2. Multi-Element Determination in Mangrove Swamp Water Samples

The results of multi-element analysis including the range and mean values of 9 mangrove swamp water (MW) samples using a MH-ICP-MS are summarized in Table 2. Among major and minor elements, chlorine (Cl), sodium (Na), magnesium (Mg), calcium (Ca), potassium (K), sulphur (S), and bromine (Br) were dominant in mangrove swamp water samples with the concentration range (mg/L) of Cl: 13–18,592; Na: 13–3722; Mg: 6.3–1542; Ca: 7.4–339; K: 0.80–331; S: 1.0–282; and Br: 0.065–112. These seven elements were present in all investigated samples. The highest concentration of these seven elements were detected in the MW 01 sample.

Lithium was detected in all mangrove swamp water samples with the concentration range of 3.8–72 µg/L, the highest concentration in this range detected from MW 01. Beryllium (Be), phosphorus (P), iron (Fe), gallium (Ga), silver (Ag), tellurium (Te), osmium (Os), mercury (Hg), and bismuth (Bi) were not detected.

No REEs were detected in mangrove swamp water samples except for (i) neodymium (Nd) in MW 01 and MW 04 at the concentration of 0.038 and 0.010 µg/L, respectively; and (ii) gadolinium (Gd) in MW 04 at the concentration of 0.0050 µg/L.

With the exception of molybdenum (Mo) and tin (Sn), no so-called Mix 2 elements (see Materials and Methods) were detected in MW samples. Molybdenum was detected in all of the samples with the concentration range of 0.057 to 8.7 µg/L. The highest concentrations of Mo were detected in the MW 01. Tin was detected only in the FW 06 sample at the concentration of 0.019 µg/L.

Silicon (Si) and boron (B) were found in all MW samples at the concentration range of 1522–14,816 and 8.6–3220 µg/L, respectively. The highest concentrations of Si and B were found in FW 06 and MW 01, respectively.

The concentrations of trace elements manganese (Mn), nickel (Ni), copper (Cu), and zinc (Zn) were detected in all samples at the range of 0.050–3.1, 0.13–19, 0.13–5.5, and 0.15–14 µg/L, respectively. The highest concentration of these four elements were found in the MW 01 sample. Cobalt (Co) was either not detected or found in the low µg/L range.

Rubidium (Rb), strontium (Sr), and uranium (U) were detected in all samples at the concentration range of 1.1–103, 58–6736, and 0.0020–1.8 µg/L, respectively. The highest concentration of these elements was found in the MW 01 sample.

Lead (Pb) was detected in all MW samples at the concentration range of 0.0070–0.77 µg/L. The highest concentration of Pb was detected in MW 01.

The total element concentration of the 9 analyzed mangrove swamp water samples was first plotted using log transformation technique to visualize full spectrum of elements.

Similar to the analysis of the freshwater samples, to the log transformed data we applied quantile normalization to generate a common unit for all element concentrations, which may be observed in Supplementary Figure S2.

### 2.3. Multi-Element Determination in Saltwater Samples around Upolu Island, Samoa

The results of multi-element analysis, including the range and mean values of 106 saltwater (SW) samples using a MH-ICP-MS, are summarized in Table 3. Among major and minor elements, chlorine (Cl), sodium (Na), magnesium (Mg), sulphur (S), potassium (K), calcium (Ca), and bromine (Br) were dominant in saltwater samples with the concentration range (mg/L) of Cl: 9690–26,870; Na: 1754–4853; Mg: 359–2686; S: 289–958; K: 116–636; Ca: 169–532; and Br: 25–74. These seven elements were present in all investigated samples. The highest concentration of chlorine and sodium were obtained in SW 090 and SW 023, respectively. Results showed that SW 038, SW 095, and SW 093 have the highest concentration of Mg, S, and Ca, respectively. The highest contents of K and Br were obtained in SW 009.

Lithium (Li) was detected in all of the samples with the concentration range of 53–201.6 µg/L with the highest concentration was detected in SW 85. Beryllium (Be), phosphorus (P), iron (Fe), yttrium (Y), cadmium (Cd), indium (In), tellurium (Te), osmium (Os), and mercury (Hg) were not detected in any sample.

Most of rare earth elements (REEs) were not detected in the saltwater samples. However, lanthanum (La) was detected in SW 012 at the concentration of 6.6 µg/L. Cerium (Ce) was detected in SW 096 and SW 032 at concentration less than 0.20 µg/L. Ytterbium (Yb) was detected in 10 SW samples in the concentration range of 0.040 to 0.48 µg/L.

Most of these so-called Mix 2 elements (see Materials and Methods) were either not detected (Hf, Ta, W, Re, and Pt) or were at very low µg/L or at the ng/L range except molybdenum (Mo), which was detected in all of the samples at the concentration range of 5.2 to 21 µg/L. The highest concentrations of Mo were detected in SW 096, SW 055, SW 009, SW 032, and SW 093.

The concentration of trace elements of manganese (Mn) and cobalt (Co) were either not detected or were at very low µg/L or at the ng/L range. Nickel (Ni) was detected in all samples at the concentration range of 5.6 to 19 µg/L. Copper (Cu) and zinc (Zn) were detected in most of the samples in the concentration ranges of 1.2–12 and 0.64–46 µg/L, respectively.

Gallium (Ga) was detected only in the SW 027 sample with the concentration of 108.9 µg/L.

Rubidium (Rb), strontium (Sr), and uranium (U) were detected in all samples at the concentration range of 43–140, 1886–7120, and 0.32–3.3 µg/L, respectively.

Lead (Pb) with the exception of two samples, was detected in all SW samples at the concentration range of 0.050–5.0 µg/L. The highest concentration of Pb was detected in SW 049 sample.

Similar to analysis of the freshwater and mangrove swamp water samples, to the log transformed data we applied quantile normalization to generate a common unit for all element concentrations, which may be visualized in Supplementary Figure S3.

**Figure 1:**
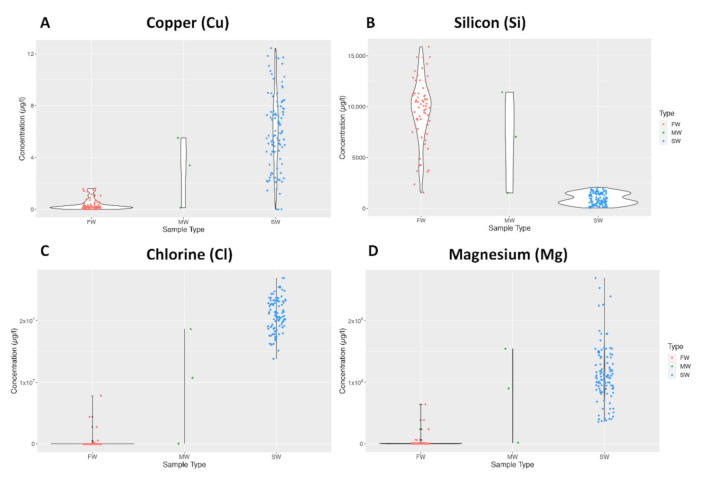
Violin plots showing the distribution of elemental data for Copper (**A**), Silicon (**B**), Chlorine (**C**), and Magnesium (**D**).

Violin plots were used to make further comparisons between freshwater (FW), saltwater (SW) and mangrove swamp (MW) water samples. The general pattern followed that of Cu concentration depicted in Figure 1A, where a relatively higher concentration of the element was present in SW. However, there were exceptions such as Si concentration in Figure 1B, where the pattern was the opposite. The ranges of the sampling groups varied significantly depending on the element, as depicted for Cl in 1C and Mg in 1D.

### 2.4. Principal Components Analysis and Elemental Correlation of All Water Samples around Upolu Island, Samoa

**Figure 2:**
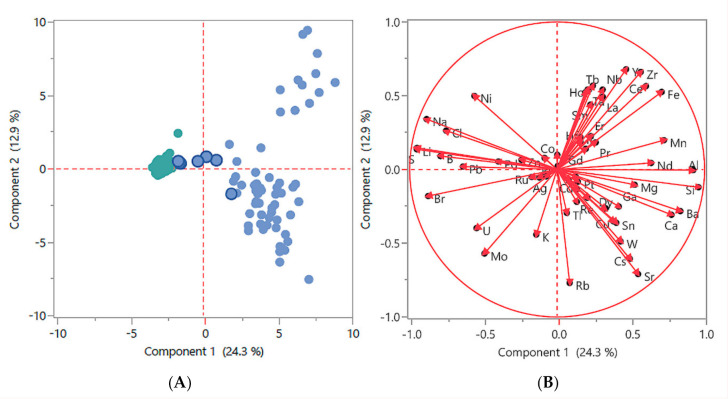
Two-dimensional graphical method of principal components analysis (PCA): employing the log transformed and quantile normalized data used in Figures S1–S3. (**A**) FW samples are represented by blue dots, saltwater samples are represented by green dots, and mangrove water samples are represented by blue dots with dark blue borders. (**B**) Elements cluster by water type, as described in the main text.

Principal components analysis (PCA) was performed on the aggregate of all water samples employed in this study. This method extracts the largest explainable variation in element concentrations into the first “component” (component 1) on the X-axis, and then the next most amount of explainable variation in component 2 on the Y-axis. This two-dimensional graphical method helps to visualize primary phenomena residing in the data that we want to understand. The two Figures, Figure 2A,B, use the log transformed and quantile normalize data employed in Supplementary Figures S1–S3. Noticeable are several samples that cluster near to the saltwater cluster in Figure 2A (blue dots with dark blue borders). These are mangrove swamp water samples (MW) that share characteristics of saltwater flowing or seeping into the swamps. In Figure 2B elements cluster by water sample types, such as the presence of bromine, sulphur, sodium, and chlorine around the 9:00–10:00 clock position for saltwater, and barium, calcium, magnesium, and silicon around the 3:00–4:00 clock position for freshwater. Elements clustering around the 1:00–2:00 clock position is in relatively low concentration in most water samples, appearing in the group of FW samples at the upper right in Figure 2A.

**Figure 3:**
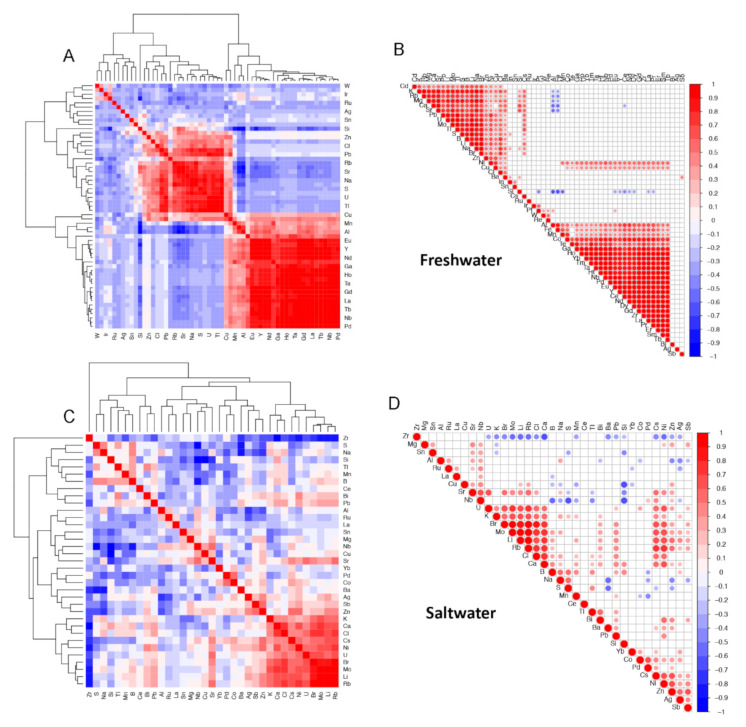
Correlation matrices (**A** and **C**) and correlograms (**B** and **D**) of elemental data for freshwater (FW, **A** and **B**) and saltwater (SW, **C** and **D**) samples. Correlation values are clustered based on similarity and their correlation coefficient values represented by a color gradient: 1 (red), 0 (white) to −1 (blue).

To complement the principal component analysis, we computed sample pairwise correlations using the elemental data in FW and SW and visually presented the results using clustered correlation matrices and correlograms. Figure 3A,B of freshwater samples, show two main clusters (highest-level connector) of elements that are significantly positively correlated. Figure 3C,D of saltwater shows less correlation structure in the seawater elements data with only one smaller cluster of covarying elements (lower-level cluster at bottom right).

### 2.5. Element Concentration Comparisons to Other Studies

The concentrations of cadmium (Cd), Cu, Ni, lead (Pb), and zinc (Zn) in freshwater samples are less than the values reported elsewhere [25].

In respect of saltwater samples, we take the view that marine organisms have adapted to “normal” values and ranges in complex oceanic systems, which we provide in Table 4 and Table 5. Apart from those values obtained for academic purposes [26], those values from government environmental and conservation councils should be particularly viewed as appropriate for aquatic life [25]. Results given in Table 4 indicate that the concentrations of Si and Sr in saltwater samples are low in some samples based on the literature reported elsewhere. However, the concentrations of other elements, such as bromine (Br), calcium (Ca), copper (Cu), magnesium (Mg), and nickel (Ni) in SW samples, are higher than typical values in about 16–64% of samples [25,26].

Element concentration profiles in mangrove swamp water (MW) samples were similar to saltwater samples as shown in Table 5. Main differences were in silicon (Si) and calcium (Ca) values, whereby Si is higher and calcium is lower in concentration than reported literature-based values [26].

### 2.6. Water Quality Assessments

Table 6 provides a series of conventional water quality assessments—salinity, pH, dissolved O_2_, oxidation-reduction potential (ORP), and nitrate that complement our ICP-MS-derived element profiles.

The pH values of FW samples were ranging from 6.6 in FW 60 to 8.3 in FW 66. The pH range of mangrove swamp water samples were from 6.9 in FW 06 to 7.8 in FW 26. These values for saltwater samples range from 7.8 in SW 021 to 8.1 in SW 077.

#### 2.6.1. Salinity and pH

The salinity values of all freshwater samples, excluding those at terminal mangrove swamps, were equal to or less than one part per thousand (‰). Mangrove swamp water (MW) samples had salinity values ranging from 1 to 34‰. Among MW samples, the highest values of 34‰, 21‰, and 15‰ were related to the MW 01, MW 02, and FW 13 samples, respectively. Salinity values for the saltwater samples were in the range of 35‰ to 54‰. Among all SW samples, 29 samples had salinity of greater than 40‰.

#### 2.6.2. Dissolved O_2_, ORP and Nitrate

Dissolved oxygen levels in waters suitable for aerobic life should not fall below 6 mg/L or roughly 80–90% saturation [25]. Our measurements were taken from mid- to late-afternoons, but not over a diurnal cycle. Nevertheless, ranges are generally accordant with excellent oxygenation except for values recorded for the Valima and Vaisigano rivers around the capital city, Apia, which both belong to the same river system, and whose values averaged 47%.

ORP on which dissolved oxygen levels and pH depend are good, typically ranging between 100 and 200 mV [32]. These values are typical of regions with waterlogged soils such as those of Samoa [33]. In general, ORP diminishes from turbulent headwaters to the slower flowing lowlands; for three rivers on which 4 measurements were taken, ORP diminished by 29 mV from the headwaters.

According to the World Health Organization (WHO) [34], unpolluted natural nitrate levels in surface waters are particularly low, at 0–18 mg/L on average. In the Samoan FW examined, only in the Falefa River estuary on the northeast coast did it reach a high level of 20 mg/L.

## 3. Discussion

### 3.1. Findings in Relation to Multi-Element Testing

**Figure 4:**
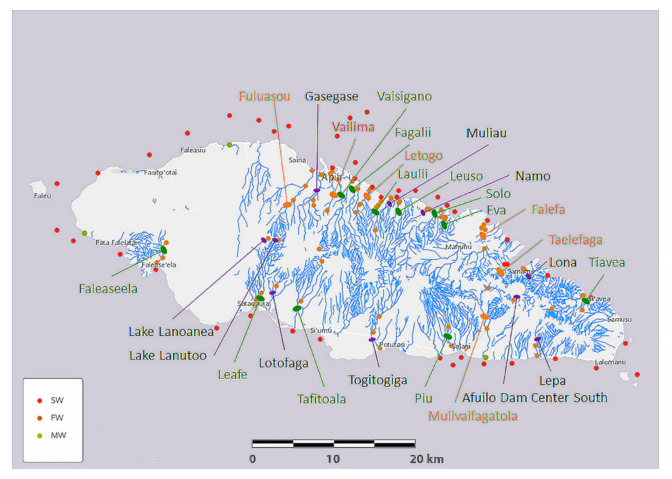
Map of Upolu Island, Samoa, showing saltwater (SW), freshwater (FW), and mangrove swamp water (MW) sampling locations (see color coded key) and river names. River names are color coded according to a ranking of urgency for mitigation given in Supplementary Table S4.

Among many parameters, elemental composition is critical to the lives of organisms. Coral reefs are distributed around most of the perimeter of Upolu Island, along which three areas have been assigned to marine protected areas (MPA), as detailed in a recent hydrographic risk assessment [35] Such MPA include Palolo Deep MPA, opposite the estuaries of Vailima, Vaisigano and Fagalii Rivers in north-central Upolu Island, Aleipata MPA that wraps around the eastern tip of Upolu Island between the Tiavea and Lepa Rivers, and the Safata MPA opposite the Leafe, Lotofaga, and Tafitoala Rivers in south-central Upolu Island (see Figure 4).

Some toxic heavy metals such as cadmium (Cd) and lead (Pb) are in low concentrations on Upolu Island (Table 4) and are unlikely the cause of harm to the Samoan population or to the environment in general.

Several alkaline earth metals are essential to life, such as strontium [36], yet all of the sample concentrations are below the typical literature-based environmental limits (Table 4). Having said this, though they are very low compared to average SW values, they are needed at extremely low amounts for organisms and are unlikely to be biolimiting.

**Figure 5:**
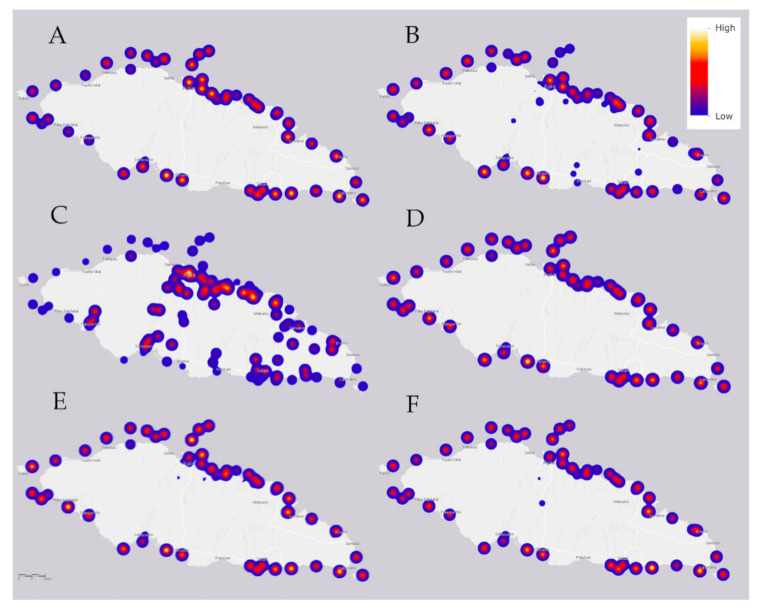
The median value for (**A**) an alkaline earth metal of magnesium (Mg); (**B**) copper (Cu); (**C**) silicon (Si); (**D**) bromine (Br); (**E**) an alkaline earth metal of calcium (Ca); and (**F**) nickel (Ni) in the various water samples of Upolu Island, Samoa. Sampling locations are shown as shades of blue, red, and yellow spots that refer to the relative heat scale at upper right. The 6 km scale is that of Figure 6.

The median value for another alkaline earth metal, magnesium (Mg; Figure 5A), is near the typical saltwater literature-based value, but 27% of seawater samples are elevated above normal, some concentrations being double the level of that which is typical in saltwater (Table 4). These high values (yellow intensities inside red circular spots) are unevenly distributed around the island, which indicates possible local toxicity due to industrial and other human activities.

As shown in Figure 5B, copper (Cu) is distributed around the coastline in moderate-to-high concentrations, 67% of the sample values increased above typical with some concentrations double that which is typical in saltwater (yellow intensities inside red circular spots) (cf. Table 3). The median value of zinc is well below usual saltwater concentration, all of the samples below typical concentration values in saltwater (Table 3). In the marine environment the major anthropogenic sources of copper, combined with transient decreases in zinc, are antifouling paints used to coat ship hulls, buoys, other underwater surfaces, and from decking, pilings and marine structures in which treated timbers are present [37,38].

Siliceous igneous rocks are the primary source of silicon on Upolu Island. Some seawater concentrations of silicon (Si; Figure 5C) are below typical concentrations values in saltwater (Table 4). However, the Si concentrations in mangrove swamps is very high (Table 2), thus this dynamic needs further investigation.

The median bromine (Br; Figure 5D) concentration is a little below usual for saltwater, but 36% of the sample values are above usual (yellow intensities inside red circular spots), reaching concentration values as much as ca. 12% above typical in saltwater (Table 4).

The median value of calcium (Ca; Figure 5E) is a little below usual in relation to those of saltwater, but 43% of the sample values are above typical (yellow intensities inside red circular spots), reaching concentration values more than 20% above typical concentration values in saltwater (Table 4). Crop farming is a potential anthropogenic source, because calcium is associated with herbicides and pesticides, as is magnesium (Mg); note the similarity in concentration maps of Mg (Figure 5A) and Ca (Figure 5E).

The median concentration of nickel (Ni; Figure 5F) is below usual compared to those of saltwater, but 16% of the samples are above typical, reaching concentration values of more than 20% above concentration values in saltwater (Table 4).

To provide circumstantial evidence for the suggestion that some elements are concentrating because of human activities, such as with herbicides and pesticides containing Br, Ca, and Mg, we list all rivers for which there are 2–4 samples taken from upstream headwaters to the lowland estuaries, and highlight those that have increasing concentrations along their lengths and that correspond with locally higher than typical saltwater values in the inner reef areas (Table 7).

The capital city of Samoa, Apia, and its surrounding suburbs are situated between The Fuluasou and Letogo Rivers, and there can be seen to be a number of elements increasing in concentration in this highly populated region of the island. The coastline east of Apia is sparsely populated, but is again more densely populated in the region occupied by the Eva and Falefa Rivers, which is also concentrating some elements in the lowlands. The southern coast is speckled with villages, most rivers having some incidences of increasing element concentrations, but particularly among the more densely populated areas around the Lepa River in the southeast and the Faleaseela River in the southwest.

The distribution of rivers in connection with urban and suburban population density variation is connected also to nearby plantations, suggesting that human activities including plant and pest management are related to the increasing concentrations of some elements.

Note that rivers associated with increasing Cl concentrations follow a different distribution pattern than those of the other elements just mentioned, with incidences of increasing Cl concentrations being more evenly distributed around the perimeter of the island (Table 7). This distribution is especially similar to that of two species of introduced myna birds inhabiting the coastal urban areas and plantations on the island. Introduced to Upolu Island, the myna bird is considered a threat to indigenous wildlife including endangered and conserved birds, and is the subject of avid extermination by use of the avicide DRC-1339 (Starlicide) [39,40]. Starlicide is acutely toxic to myna birds, and though it has other specific avian targets, including gulls, it has a wide toxic net to other families of birds and to some mammals, and is acutely toxic to aquatic invertebrates and some fish [41]. While aquatic environmental toxicity testing has only been performed in freshwater settings, marine invertebrates and fish are also likely susceptible.

Except for three REEs of cerium (Ce), lanthanum (La), and gadolinium (Gd), little is known on the health effects of these elements [42]. The concentration of 10 REEs of La, Ce, neodymium (Nd), samarium (Sm), europium (Eu), Gd, dysprosium (Dy), erbium (Er), ytterbium (Yb), and lutetium (Lu) have been measured in 31 samples from 15 rivers (water samples were collected during a series of transects along the Connecticut, Delaware, Mullica (USA), Amazon, and Tamar (UK) river estuaries) [43]. The concentrations of these 10 elements in two freshwater samples, FW 55 and FW 56 in downstream waters of the Tiavea River, were generally within the range but at the highest levels reported in that study [43].

We note that correlations of element concentration values are clustered (Figure 3). Among the FW samples, elements described in this study as potentially arising in concentrations due to human activity—Ba, Br, Ca, Cu, Mg, Ni, Si, Sr, and Cl—cluster together and are positively correlated. This pattern is only partially borne out in the SW samples in which Br, Ca, Ni, and Cl are clustered and positively correlated, while Sr is correlated to a lesser degree to this group of elements in a connected supercluster.

The small clusters from both the freshwater and saltwater samples in Figure 3 contain a number of positively correlated elements that may be naturally occurring ionic compounds, such as lithium bromate/bromide, lithium chlorite/chlorate, potassium bromate, rubidium bromate/bromide rubidium chloride, and strontium chloride.

This is the first time such statistical analyses as these have been performed on such a comprehensive number of inorganic elements from environmental samples, and thus this it is at best a first pass at interpreting and understanding the results.

### 3.2. Salinity and pH

Salinity in ocean water derives mainly from sodium chloride and is measured in parts per thousand (‰). Salinity of South Pacific water is in equilibrium at around 35–36‰ [44]. During a recent survey of salinity around American Samoa at an average depth of 11.45 m, the median salinity was 34.63‰ with a range of only 1.39‰ [45]. Around Upolu Island, ca. 30% of all saltwater samples are well above the South Pacific norm, in the range of 52–54‰ (Figure 4). A total of 15 water samples was collected at three different sites of the Vaisigano river in Samoa and the pH range of 8.2–8.5 was reported [17]. The pH range of our investigated various water samples show relatively lower pH.

### 3.3. Dissolved O_2_, ORP, and Nitrate

Generally good dissolved O_2_ levels and ORP values, together with low nitrate levels indicates that Upolu is not causing eutrophication of the surrounding sea (i.e., excessive nutrients that drive the growth of phytoplankton and other algae and reductions of dissolved oxygen), which would otherwise pose a threat to aquatic life.

### 3.4. Findings in Relation to Human Health and the Environment

A 1994 survey of South Western Pacific waters cited overfishing, pollution, eutrophication, and erosion leading to sedimentation as a major threat to lagoons and coral reefs [15]. A more recent report on the status of the coral reefs around Upolu Island speculate that a series of tropical cyclones and the 2009 tsunami have exacerbated these conditions leading to substantial coral reef decline [16]. Missing from these studies, however, is a comprehensive discovery-based system for identifying the fine-grained detail laying at the foundation of threats to the coral reefs. Because coral reefs are a vital hub in the global interdependent network of ecosystems, perturbations to this network should be visible in all areas of the ecosystem such as may be expressed on nearby terrestrial environments in relation to human health issues.

The incidence of obesity in the island state of Samoa is among the highest in the world [46], which includes all of the well-recognized concomitant complications of the condition. Locally high levels of magnesium (Figure 5A), bromine (Figure 5D), and calcium (Figure 5E) are putatively connected to the widespread use of 71 herbicide and pesticide chemicals on Upolu Island, which have endocrine effects suggested to be contributing to endemic obesity [47]. For instance, the nonuniformity of bromine suggests that it may be supplemented by water and waste treatment, as a fungicide, and as an agricultural pesticide against snails [48]. The primary means of exposure to herbicides and pesticides is by dermal exposure [49], and airborne incidences and exposure while swimming and bathing in contaminated waters are likely responsible.

Olivine basalts dominate the lithology of South Pacific islands such as Upolu Island [50]. The chemistry of olivine is (Mg,Fe)_2_SiO_4_, thus magnesium levels reported in this study could derive from the local rock. Indeed, magnesium is reported to be high in soils of Taro farms on the Island [51]. However, Taro farms are susceptible to numerous pests and pesticide control measures [52], leaving open the sourcing of the element. Iron levels from the freshwater sources examined should similarly be elevated because of olivine chemistry, but it was typically so low as to be not detected.

The herbicides and pesticides that include magnesium, calcium, and chlorine are highly toxic to aquatic life. Thus, there is an intimate connection between their role in the environment on both human and aquatic health. This is a major justification for the employ of the comprehensive discovery-based system employed in this study on Upolu Island. Magnesium, copper, calcium, and nickel are ubiquitously or locally high around Upolu Island. These increases in concentrations of accumulated heavy metals—even those essential to life—are associated with coral reef decline [53]. In a further example of the relationship between assessments of the environment and human health, we note that Ni and the Cl-containing avicide, are associated with contact dermatitis in humans [54], which may increase the potential for additional vulnerabilities and exacerbation of insults through the skin. These associations between metal environmental contamination and human and aquatic health require study, analysis, and confirmation on Upolu Island.

Among the elements essential to life, silicon and strontium are of particular interest [19,55]. Silicon is essential for growth of unicellular algae (diatoms) that support marine food webs in coastal areas and islands and for the construction of stony corals, however the input from freshwater sources appears to be controlled to some extent at mangrove swamps, rendering an insufficiency in places and potentially limiting the growth of coral reefs. The contrast between high freshwater sources and low seawater concentrations in some samples may also suggests that excess inputs due to farming activities and/or climate effects may be dumping silica from the rivers into the sea [56,57], which falls from surface waters to the sea floor and obscuring the coral reef surface, thus decreasing the coral reefs’ ability to thrive. The fate of river-born silicon upon entering Upolu Island lagoons, requires further study.

Strontium is essential for the growth and construction of stony corals [36], yet the concentrations are very low around Upolu Island. There is presently no explanation for how strontium can be sequestered to such levels.

**Figure 6:**
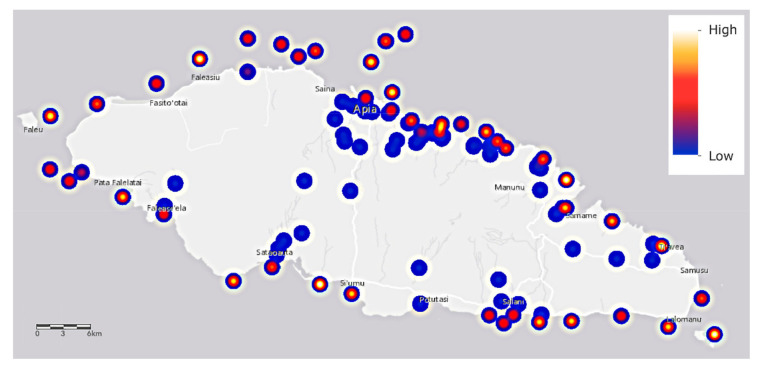
The salinity values of saltwater samples around Upolu Island, Samoa. Sampling locations are shown as shades of blue, red, and yellow spots that refer to the relative heat scale at upper right.

The salinity (Figure 6) of about one third of all saltwater samples is higher than typical for coral organisms whose salinities range from 32 to 42 parts per thousand [58]. Some corals are specifically adapted to very saline +50% conditions, such as those of the Red Sea, and it is suggested that their tolerance of such high values may be higher than corals in other oceans [59]. We are concerned that the high levels observed in some locations may then be lethal to coral organisms of Upolu Island. Values should be checked against historical records.

Finally, a feature of some element concentration maps is the increase in river elemental concentrations from the interior of Upolu Island to the coastal regions (Table 7). This is an indication of cumulative increases due to human activities whose related chemicals are as likely to be harmful to humans as they are to aquatic life.

To address factors deleterious to human, economic and recreational activities and coral reef health, the Government of Samoa may consider the following recommendations:

1. Mitigating environmental harm caused by use of herbicide and pesticide chemicals through alternative pest management, such as biological control alternatives and hunting incentives. A chemical survey of Upolu Island is required to confirm the source of some elements to the local lithology and/or chemical use;
2. Conducting specific investigations into strontium, and silicone deficiencies. Historically this may be investigated by sampling coral skeletons retrieved at known dates in the past;
3. Investigating causes of salinity variability of the inner reef and reef zone. Multivariate analyses of historical weather and ocean data with present day sampling through seasons is warranted;
4. Future studies need to be performed drawing on the results of this research to see if there are medical cluster effects that correlate to water quality as revealed in Figure 4.

To assist with establishing mitigation priorities, we generated a table of relative urgency (Supplementary Table S4). Based on freshwater and saltwater element concentrations, which putatively relate to harmful chemicals, and on elevated salinity levels reported in this study, we subjectively ranked the urgency for mitigation and accordingly color coded the river names in Figure 4.

## 4. Materials and Methods

### 4.1. Study Area and Sampling

Sampling of the freshwater and saltwater resources of Upolu Island took place through December 1–15, 2019. Five field teams were composed of personnel of the Samoan Ministry of Natural Resources and Environment and the Faculty of Science, National University of Samoa. “Land” teams of 4 people were dispatched to each of northern, eastern, southern, and western portions of the Upolu Island. A “Boat” team consisting of two divers and a drone operator circumnavigated the island, docking locally at the end of each day, being picked up to return home, and driving to the last destination each morning to begin sampling along the next coastal segment. 50 mL sampling tubes were used to acquire all water samples from the surface of each of a river’s headwater and its mouth and location(s) in between, from lakes, and from the inner reef, reef, and outer reef locations that included depths to 30 m that capture water in contact with shallow water coral reefs.

A total of 67 freshwater, 106 saltwater, and 9 mangrove swamp water samples (6 of these samples were marginal to freshwater samples and labeled as such for some analyses) were collected. Freshwater samples were collected from 24 rivers, 2 volcanic lakes, and 1 dam, saltwater samples were collected from 45 inner reef, reef, and outer reef locations around Upolu Island, including each river estuary, and collected from 3 mangrove swamp locations as shown in Figure 4.

All samples were stored in a refrigerator at about 4 °C prior to analysis.

### 4.2. Materials

Suprapur^®^ 65% HNO_3_ (analytical-reagent grade, Merck, Darmstadt, Germany), de-ionized ultrapure water (18.2 MΩ cm) (ELGA, Purelab^®^Ultrapure Water Purification Systems, Lane End, Buckinghamshire, UK), and National Institute of Standards and Technology (NIST) standard reference material^®^ (SRM)1640a (trace elements in natural water, NIST, Gaithersburg, MD, USA) were used in this study. All water samples were collected into 50 mL HDPE conical centrifuge tubes previously cleaned with 2% (*v*/*v*) Suprapur^®^ 65% HNO_3_ to eliminate trace contaminants that would otherwise contribute to the element composition.

Following single and multi-element standards were used to prepare calibration standard solutions containing four internal standards of ^6^Li (enriched lithium), Ge, Rh, and Th.

Single element standards: twelve single element standards of Cl, Os, K, Si, S, P, Na, Mg, Ca, Ti, Br, Hg, ^6^Li, Ge, Rh, and Th all purchased from Inorganic Ventures (Christiansburg, VA, USA) at the concentration of 100 or 1000 µg/mL were used to prepare single element, multi-element calibration standards, and the mix of internal standards.

Multi-element standards: four multi-element standards of (i) Merck VI (Certipur^®^ Certified Reference Material ICP multi-element standard VI: Merck KGaA, Darmstadt, Germany) contains 30 elements of Al, As, Ba, Be, Bi, B, Cd, Ca, Cr, Co, Cu, Ga, Fe, Pb, Li, Mg, Mn, Mo, Ni, K, Rb, Se, Ag, Na, Sr, Te, Tl, U, V, and Zn at different concentrations ranged from 10 to 1000 mg/L; (ii) Mix 1 (Trace cert^®^ Sigma-Aldrich Production GmbH, Buchs, Switzerland) contains 33 elements similar to Merck VI solution excluding Mo and U but instead has additional five elements of Cs, In, P, Si, and S at the concentration of about 10 mg/L; (iii) Mix 2 (Trace cert^®^ Sigma-Aldrich Production GmbH, Buchs, Switzerland) contains 17 elements of Sb, Ge, Au, Hf, Ir, Mo, Nb, Pd, Pt, Re, Rh, Ru, Ta, Sn, Ti, W, and Zr at the concentration of 10.01 mg/L; and iv) Mix 3 (Trace cert^®^ Sigma-Aldrich Production GmbH, Buchs, Switzerland) contains 16 rare earth elements (REEs) of Ce, Dy, Er, Eu, Gd, Ho, La, Lu, Nd, Pr, Sm, Sc, Tb, Tm, Yb, and Y at the concentration of about 10 mg/L, all known as certified reference materials (CRMs), were used to prepare multi elements calibration standards.

### 4.3. Instrumentation

Total element concentrations of various water samples were measured using Mattauch–Herzog-inductively coupled plasma-mass spectrometry (MH-ICP-MS) SPECTRO MS (SPECTRO Analytical Instruments GmbH, Kleve, Germany) based on our published method with further modifications [12]. Most of freshwater samples with salinity of ≤1 part per thousand (‰) were undiluted introduced to the nebulizer of MH-ICP-MS by a Teledyne autosampler (Model ASX-560, Teledyne CETAC Technologies, Omaha, NE, USA) while the saltwater samples were diluted by a factor of 40–50 based on their high salinity. Salinity measurements were carried out using a digital seawater refractometer (Milwaukee, MA887, Romania). All calibration standards and samples contain 2% (*v*/*v*) Suprapur^®^ 65% HNO_3_ and internal standards of ^6^Li, Ge, Rh, and Th at the concentration of 100, 50, 20, and 20 µg/L, respectively. In order to take the variability of the ICP-MS conditions into account while performing the water measurements, these four internal standards were used for various mass ranges. Isotopes of ^6^Li, ^72^Ge, ^103^Rh, and ^232^Th were selected and used for ^7^Li–^44^Ca, ^45^Sc–^89^Y, ^90^Zr–^159^Tb, and ^163^Dy–^238^U mass ranges, respectively. Limit of detection, correlation coefficient of calibration plots for each element, and selected isotopes for the determination of elemental concentration in various water samples of Upolu Island, Samoa are summarized in Supplementary Table S5. MH-ICP-MS was optimized and tuned each time for its performance within the entire breadth of the chemical periodic table, i.e., from Li to U using 20 µg/L of Merck VI multi-element standard solution to achieve maximum sensitivity. To check the repeatability of analyses, various single and multi-element standard solutions and NIST SRM-1640a Trace Elements in Natural Water (see Supplementary Table S6) as well were used in the beginning and after every five samples in each set of samples run. To promote comparative measurements, samples were left to settle for one week prior to ICP-MS measurements and the autosampler probe adjusted to draw from above the 1 mL mark on the 15 mL tube.

The physical parameters were determined using a handheld YSI ProDSS Multimeter (YSI Inc., USA) with attachments capable of measuring, dissolved oxygen (DO, polarographic sensor, accuracy: ±1%), oxidation-reduction potential (ORP, platinum button sensor, accuracy: ±20 mV) and nitrate (NO_3_^−^, ion selective electrode, accuracy: ±2 mg/L). Data were acquired in freshwater and mangrove swamp water for DO, ORP, and NO_3_^−^ and in saltwater samples for salinity, DO, and ORP.

Some operation conditions of MH-ICP-MS is summarized in Table 8.

### 4.4. Plots and Statistical Analysis

The raw data files were cleaned and concentration values for elements that were detected in none (Sc, Ti, V, Cr, As, Se) or in only a few (Be, P, Lu, Os, Au, Hg) samples were filtered out. Undetected reads were imputed values of 0 and the data was log transformed. The SAS/JMP software (SAS Institute) was used to quantile normalize the data by sample, to generate the heat maps (see Supplementary Figures S2 and S3), and to carry out the principal component analysis (Figure 2). Quantile normalization is based on density-adjusted rank ordering and makes distributions identical in statistical properties [60]. The method equilibrates all ranks by assigning each measure or data point the arithmetical mean value across water samples for each rank (i.e., the highest value in all water samples becomes the mean of the highest values, the second highest value becomes the mean of the second highest values, and so on).

Plots for Figure 4, Figure 5 and Figure 6 were created using the ArcGIS online platform. The hydrology layer, which depicts the rivers of Samoa was provided by the Samoan ministry in the form of a zipped file containing dBASE, ProJect, Quantum GIS, SHX and shapefile components. These files were converted using ArcGIS Desktop for online use. Raw elemental reads were uploaded for use and samples SW105_75435, SW106_76685, SW103_73578, SW104_73752 were removed as they did not contain GPS data. The smart mapping function was used to create heat maps for the individual elements, weighting the data to calculate a depiction of density along a dark blue to yellow color gradient. The area of influence was set to 25% to enable the optimum visualization of the sampling sites, especially those in closer geographical proximity, and the transparency parameter was set to 0% to maximize contrast against the basemap.

## 5. Conclusions

A comprehensive study was performed in the mangrove swamp, freshwater, and saltwater environmental resources of Upolu Island, Samoa to analyze up to 69 elements in each sample simultaneously in a single run using Mattauch–Herzog-inductively coupled plasma mass spectrometer (MH-ICP-MS).

In our study of the biosecurity of Upolu Island freshwater and saltwater environmental resources we have identified several environmental challenges that individually or, more likely, cumulatively, may explain problems that require mitigation. The contributions of metals to the coastline of Upolu Island require attention. Copper from antifouling paints, an apparently ubiquitous feature of marine infrastructure, should be carefully considered by local authorities. Identification of specific sources of metal contamination and their reduction using new or improved technologies and manufacturing processes are needed.

Strontium is essential to the lives of corals and in surprisingly low concentrations around Upolu Island. These insufficiencies require investigation. Historically, this may be investigated by sampling coral skeletons retrieved at known dates in the past.

Herbicide and pesticide chemical use on Upolu Island is widespread and magnesium, bromine, and calcium are the likely signatures. These chemicals have endocrine effects that may contribute to endemic obesity and they are also highly toxic to aquatic life. Mitigation efforts may include alternative pest management, such as biological controls.

Salinity of coastal waters should be investigated. If increased salinity is found to be recent, it may be due to climate induced decreased freshwater flow into the sea and/or evaporation from relatively sequestered landward sides of coral reefs. Causes of salinity variability of the inner reef and reef zone require investigation, and multivariate analyses of historical weather and ocean data with present day sampling through seasons is warranted.

Our comprehensive discovery-based system has revealed several potential issues that may relate to observed coral reef decline. These include putative element abundances due to chemical toxicities, which is the focus of this communication, in addition to microbial diversity and the distribution of microplastics in the environment that individually, or more likely cumulatively, have caused the deterioration of Upolu’s coral reefs.

## Figures and Tables

**Table 1:** Elemental composition in 66 freshwater samples (FW) from various rivers and lakes.

	Concentration (µg/L)			Concentration (µg/L)			Concentration (µg/L)	
Element	Range	Mean	Element	Range	Mean	Element	Range	Mean
Li	<LoD ^a^–1.5	<1.5	Br	5.2–853	33	Eu	0.0030–0.034	0.012
Be	<LoD	<LoD	Se	0.030–17	0.99	Gd	0.0030–0.095	0.026
B	0.40–39	5.1	Rb	0.16–8.5	2.8	Tb	0.0040–0.012	0.0083
Na	1277–90,863	6171	Sr	7.7–252	65	Dy	0.0040–0.080	0.017
Mg	893–19,571	6658	Y	0.0030–0.41	0.094	Ho	0.011–0.012	0.012
Al	1.3–181	17	Zr	0.0020–2.2	0.20	Er	0.0040–0.044	0.014
Si	1564–15,887	9493	Nb	0.0050–0.31	0.050	Tm	0.0020–0.0030	0.0025
P	<LoD–6.7	<6.7	Mo	0.0040–0.29	0.065	Yb	0.0060–0.036	0.025
S	<LoD–258	<258	Ru	0.0070–0.0090	0.0079	Lu	0.0020–0.0030	0.0025
Cl	8.9–133,023	5821	Ag	0.0060–0.021	0.014	Hf	0.0040–0.053	0.023
K	74–3909	803	Pd	0.0080–0.062	0.034	Ta	0.017–0.021	0.019
Ca	915–24,663	7333	Cd	0.0080–0.019	0.012	W	0.0020–0.039	0.0088
Ti	0.018–105	6.1	In	<LoD	<LoD	Re	0.0050–0.0090	0.0071
V	0.27–7.4	2.9	Sn	0.0030–0.017	0.0078	Os	<LoD	<LoD
Cr	0.18–2.4	0.57	Sb	0.0030–0.24	0.030	Ir	<LoD–0.0050	<0.0050
Fe	0.20–848	110	Te	0.012–0.16	0.088	Pt	0.0060–0.014	0.010
Mn	0.0020–42	1.3	Cs	0.0010–0.052	0.020	Au	<LoD	<LoD
Co	0.0030–0.34	0.053	Ba	1.1–22	4.2	Hg	<LoD	<LoD
Ni	0.0060–5.2	0.52	La	0.0020–0.34	0.075	Tl	0.0010–0.19	0.016
Cu	0.013–1.6	0.34	Ce	0.0020–1.0	0.17	Pb	0.0060–0.11	0.026
Zn	0.024–4.7	1.3	Pr	0.0020–0.090	0.021	Bi	0.0030–0.0060	0.0041
Ga	0.010–0.33	0.041	Nd	0.0030–0.50	0.064	U	0.0010–0.095	0.0094
As	0.0010–0.46	0.12	Sm	0.0070–0.10	0.053			

^a^ < LoD: below the limit of detection.

**Table 2:** Elemental composition in 9 mangrove swamp water samples (MW) from various swamps.

	Concentration (µg/L)			Concentration (µg/L)			Concentration (µg/L)	
Element	Range	Mean	Element	Range	Mean	Element	Range	Mean
Li	3.8–72	26	Sr	58–6736	1872	Gd	<LoD–0.0050	<0.0050
Be	<LoD ^a^	<LoD	Y	<LoD	<LoD	Tb	<LoD	<LoD
B	8.6–3220	912	Zr	<LoD–0.0070	<0.0070	Dy	<LoD	<LoD
Na	12,566–3,722,473	1,030,848	Nb	<LoD	<LoD	Ho	<LoD	<LoD
Mg	6315–1,541,911	429,281	Mo	0.057–8.7	2.4	Er	<LoD	<LoD
Al	2.1–6.2	3.6	Ru	<LoD	<LoD	Tm	<LoD	<LoD
Si	1522–14,816	9490	Ag	<LoD	<LoD	Yb	<LoD	<LoD
P	<LoD	<LoD	Pd	<LoD	<LoD	Lu	<LoD	<LoD
S	1038–282,435	104,368	Cd	0.043–0.45	0.20	Hf	<LoD	<LoD
Cl	13,294–18,592,427	5,027,907	In	<LoD–0.0050	<0.0050	Ta	<LoD	<LoD
K	808–331113	91,270	Sn	0.0040–0.019	0.012	W	<LoD–0.0040	<0.0040
Ca	7429–339042	99,982	Sb	<LoD–0.0030	<0.0030	Re	<LoD	<LoD
Fe	<LoD–0.20	<0.20	Te	<LoD	<LoD	Os	<LoD	<LoD
Mn	0.050–3.1	0.98	Cs	0.0090–0.33	0.10	Ir	<LoD	<LoD
Co	0.0060–0.024	0.016	Ba	4.0–22	10	Pt	<LoD	<LoD
Ni	0.13–19	5.9	La	<LoD	<LoD	Au	<LoD	<LoD
Cu	0.13–5.5	1.6	Ce	<LoD	<LoD	Hg	<LoD	<LoD
Zn	0.15–14	4.44	Pr	<LoD	<LoD	Tl	0.0080–0.67	0.24
Ga	<LoD	<LoD	Nd	0.010–0.038	0.024	Pb	0.0070–0.77	0.23
Br	65–112,023	30,985	Sm	<LoD	<LoD	Bi	<LoD	<LoD
Rb	1.7–103	30	Eu	<LoD	<LoD	U	0.0020–1.8	0.46

<LoD ^a^: below the limit of detection.

**Table 3:** Elemental composition in 106 saltwater samples from various locations listed below.

	Concentration (µg/L)			Concentration (µg/L)			Concentration (µg/L)	
Element	Range	Mean	Element	Range	Mean	Element	Range	Mean
Li	53–202	145	Sr	1886–7120	5075	Gd	<LoD	<LoD
Be	<LoD ^a^	<LoD	Y	<LoD	<LoD	Tb	<LoD	<LoD
B	1340–3852	2823	Zr	0.040–0.25	0.13	Dy	<LoD	<LoD
Na	1,753,860–4,852,950	3,817,392	Nb	0.040–0.24	0.18	Ho	<LoD	<LoD
Mg	359,400–2,686,320	1,123,246	Mo	5.2–21	16	Er	<LoD	<LoD
Al	1.2–24	7.5	Ru	0.080–1.0	0.31	Tm	<LoD	<LoD
Si	44–2060	956	Ag	0.040–0.72	0.33	Yb	0.040–0.48	0.19
P	<LoD	<LoD	Pd	0.080–0.80	0.33	Lu	<LoD	<LoD
S	288,820–958,080	680,827	Cd	<LoD	<LoD	Hf	<LoD	<LoD
Cl	9,690,340–26,869,680	20,737,672	In	<LoD	<LoD	Ta	<LoD	<LoD
K	115,960–635,742	351,765	Sn	0.040–0.24	0.11	W	<LoD	<LoD
Ca	168,880–532,520	374,717	Sb	0.040–0.48	0.25	Re	<LoD	<LoD
Fe	<LoD	<LoD	Te	<LoD	<LoD	Os	<LoD	<LoD
Mn	0.10–3.8	1.6	Cs	0.080–0.52	0.31	Ir	<LoD	<LoD
Co	0.050–1.6	0.55	Ba	2.6–6.7	5.1	Pt	<LoD	<LoD
Ni	5.6–19	11	La	<LoD–6.6	<6.6	Au	<LoD–0.20	<0.20
Cu	1.2–12	6.2	Ce	0.12–0.16	0.14	Hg	<LoD	<LoD
Zn	0.60–45	16	Pr	<LoD	<LoD	Tl	0.050–2.4	0.33
Ga	<LoD–109	<109	Nd	<LoD	<LoD	Pb	0.050–5.0	0.98
Br	25,160–74,080	62,331	Sm	<LoD	<LoD	Bi	0.040–0.48	0.21
Rb	43–140	111	Eu	<LoD	<LoD	U	0.32–3.3	2.6

<LoD ^a^: below the limit of detection.

**Table 4:** Selected element concentrations of freshwater and saltwater samples: a comparison between literature-based values and Upolu Island water sources. U.S. Environmental Protection Agency maximum concentration limits (mg/L) for drinking water are given for FW samples in parentheses [27].

		Concentration (µg/L)			Concentration (µg/L)	
Element	FW ^a^ Values from Literature	Median	Range	SW ^b^ Values from Literature	Median	Range
Ba	× ^c^ (2)	3.5	1.1–22	4–21 [26,28]	5.2	2.6–6.7
Br	×	33	5.2–853	67,116 [26]	65,600	25,160–74,080
Ca	×	6732	915–24,663	412,824 [26]	372,560	168,880–532,520
Cd	0.2–2.0 [25](0.005)	0.01	0.0080–0.019	2 [25]	<LoD	<LoD
Cu	0.2–5 [25] (1.3)	0.18	0.013–1.6	5 [25]	6.16	1.2–12
Hg	0.1 [25] (0.002)	<LoD ^d^	<LoD	0.1 [25]	<LoD	<LoD
Mg	×	6082	893–19,571	1,293,292 [26]	1,091,100	359,400–2,686,320
Ni	15–150 [25]	0.17	0.0060–5.2	15 [25]	11	359,400–2,686,320
Pb	1–5 [25] (15)	0.01	0.0060–0.11	5 [25]	0.22	0.019–0.77
Si	×	10,001	1564–15,887	228–2809 [26,29]	868	44–2060
Sr	×	55	7.7–252	7666–7885.8 [26,30]	4920	1886–7120
Zn	5–50 [25] (5)	0.74	0.024–4.7	0.013–50 [25,31]	17	0.60–46

FW ^a^: Freshwater samples; SW ^b^: Saltwater samples; × ^c^: not reported; <LoD ^d^: below the limit of detection.

**Table 5:** Selected element concentrations of Upolu Island Mangrove Swamps water samples compared with literature-based values.

Element	Values from Literature (µg/L)	Concentration (µg/L)	
		Median	Range
Ba	13.7 [26]	10	4.0–22
Br	67,116 [26]	30,985	64.8–112,023
Ca	412,824 [26]	99,982	7429–339,042
Cu	5 [25]	1.6	0.13–5.5
Mg	1,293,292 [26]	429,281	6315–1,541,911
Ni	15 [25]	5.9	0.13–19
Si	2809 [26]	9490	1522–14,816
Sr	7886 [26]	1872	58–6736
Zn	50 [25]	4.4	0.15–14

**Table 6:** Water quality parameters measured in freshwater (FW), saltwater (SW), and mangrove swamp water (MW) samples of Upolu Island, Samoa.

	FW			SW			MW		
Parameters	Mean	Median	Range	Mean	Median	Range	Mean	Median	Range
**Salinity (‰)**	0.7	1	<1–1	41	37	13–54	9.7	3	1–34
**pH**	7.5	7.5	6.6–8.3	7.9	7.9	7.8–8.1	7.5	7.5	6.9–7.8
**Dissolved O_2_ (ppm)**	92	98	26–130	98	96	84–120	98	99	90–104
**ORP ^a^ (mV)**	152	152	76–191	128	132	88–176	137	145	19–186
**Nitrate (mg/L)**	0.61	0.08	0.01–21	NA ^b^	NA	NA	4.7	0.12	0.02–21

ORP ^a^: Oxidation-Reduction Potential; NA ^b^: Not analyzed.

**Table 7:** Selected elements of Upolu Island freshwater river samples. Rivers having both upstream and downstream samples are listed in order of their location beginning from the most northwestern river in a clockwise direction around the island to the most south western location (Figure 4). A red cell indicates that an element has increased in concentration along the length of the river.

Element	Ba	Br	Ca	Cu	Mg	Ni	Si	Sr	Cl
River									
North Coast									
Fuluasou									
Gasegase									
Vailima									
Vaisigano									
Fagalii									
Letogo									
Laulii									
Leuso									
Namo									
Solo									
Eva									
Falefa									
Taelefaga									
Lona									
Tiavea									
									
**South Coast**									
Lepa									
Mulivaifagatola									
Piu									
Togitogiga									
Tafitoala									
Lotofaga									
Leafe									
Faleaseela									

**Table 8:** Operation conditions of MH-ICP-MS.

**Masses**	From 6 (Li) to 238 (U) covering 69 elements
**Integration Parameters**	Total time: 20.0 s; Base interval: 10 ms; Mode: Threshold
**RF Power**	1465 W
**Sampler and Skimmer cones**	Ni (ICPMS Cones Limited, Tarvin Nr. Chester, Cheshire, UK)
**Spray Chamber**	Cyclonic (Spectro/AMETEK, Mahwah, NJ, USA)
**Torch Position (X:Y:Z)**	(−2.2 mm: (2.0 mm:0.2 mm)
**Plasma Argon Flow Rate**	12.0 L·min^−1^
**Auxiliary Argon Flow Rate**	2.40 L·min^−1^
**Nebulizer Argon Flow Rate**	0.91–0.93 L·min^−1^
**Nebulizer**	SeaSpray (Glass Expansion, Pocasset, MA, USA)

## Supplementary Materials

Supplementary materials are available online. Figure S1: Log transformed and quantile normalized element concentration data in freshwater samples. Trace concentrations range between dark blue (not detected) and dark red (maximum concentration). The legend to the right shows the color scale of the data representing arbitrary (normalized) values from low (blue) to high (red) and not absolute element concentrations. The element symbol is above its own colored column. Each row in the plot is an individually collected freshwater (FW) sample, and given in the left margin of each row is its sample number for associating with rivers and villages of Upolu Island (see Supplementary Table S1), Figure S2: Log transformed and quantile normalized element concentration data in mangrove swamp water samples. Trace concentrations range between dark blue (not detected) and dark red (maximum concentration). The legend to the right shows the color scale of the data representing arbitrary (normalized) values from low (blue) to high (red) and not absolute element concentrations. The element symbol is above its own colored column. Each row in the plot is an individually collected mangrove swamp water (MW) sample, and given in the left margin of each row is its sample number for associating with rivers and villages of Upolu Island (see Supplementary Table S2), Figure S3: Log transformed and quantile normalized element concentration data in 106 saltwater samples and 3 MW samples. Trace concentrations range between dark blue (not detected) and dark red (maximum concentration). The legend to the right shows the color scale of the data representing arbitrary (normalized) values from low (blue) to high (red) and not absolute element concentrations. The element symbol is above its own colored column. Each row in the plot is an individually collected saltwater (SW) sample, and given in the left margin of each row is its sample number for associating with rivers and villages of Upolu Island (see Supplementary Table S3), Table S1: Freshwater (FW) sample codes: associating with rivers and villages of Upolu Island, Samoa, Table S2: Mangrove Swamp water (MW) sample codes: associating with rivers and villages of Upolu Island, Samoa, Table S3: Saltwater (SW) sample codes: associating with rivers and villages of Upolu Island, Samoa, Table S4: Mitigation ranking by village and river. Based on freshwater and saltwater element concentrations, which relate to harmful chemicals, and on elevated salinity levels reported in this study, we subjectively ranked the urgency for mitigation and color coded accordingly the river names in Figure 4 as illustrated in this scheme, Table S5: Limit of detection, correlation coefficient of calibration plots for each elements, and selected isotopes for the determination of elemental concentration in various water samples of Upolu Island, Samoa.
