# Supplementary material for: MH-ICP-MS Analysis of the Freshwater and Saltwater Environmental Resources of Upolu Island, Samoa
Source: Molecules. 2020 Oct 22;25(21):4871. doi: 10.3390/molecules25214871 (PMC7659969; doi:10.3390/molecules25214871)
Supplement: Supplementary file 1 [file molecules-25-04871-s001.pdf]

Supplementary Materials (SM)

MH-ICP-MS Analysis of the Freshwater and Saltwater Environmental Resources of Upolu Island, Samoa

Sasan Rabieh [1,*], Odmaa Bayaraa [2], Emarosa Romeo [3], Patila Amosa [4], Khemet Calnek [1], Youssef Idaghdour [2], Michael A. Ochsenkühn [5], Shady A. Amin [5], Gary Goldstein [6] and Timothy G. Bromage [1,7,*]

[1] Department of Molecular Pathobiology, New York University College of Dentistry, 345 East 24th Street, New York, NY 10010, USA; khemet.calnek@nyu.edu (K.C.)

[2] Environmental Genomics Lab, Biology Program, Division of Science and Mathematics, New York University Abu Dhabi, Saadiyat Island, PO Box 129188, Abu Dhabi, United Arab Emirates; ob733@nyu.edu (O.B.); youssef.idaghdour@nyu.edu (Y.I.)

[3] Hydrology Division, Ministry of Natural Resources and Environment, Level 3, Tui Atua Tupua Tamasese Efi Building (TATTE), Sogi., P.O. Private Bag, Apia, Samoa; emarosa.romeo@mnre.gov.ws (E.R.)

[4] Faculty of Science, National University of Samoa, PO Box 1622, Apia, Samoa; p.amosa@nus.edu.ws (P.A.)

[5] Marine Microbial Ecology Lab, Biology Program, New York University Abu Dhabi, Saadiyat Island, PO Box 129188, Abu Dhabi, United Arab Emirates; mao13@nyu.edu (M.A.O.); samin@nyu.edu (S.A.A.)

[6] College of Dentistry, New York University, 345 East 24th Street, New York, NY 10010, USA; gary.goldstein@nyu.edu (G.G.)

[7] Department of Biomaterials, New York University College of Dentistry, 345 East 24th Street, New York, NY 10010, USA

* Correspondence: sasan.rabieh@nyu.edu (S.R.); tim.bromage@nyu.edu (T.G.B.); Tel.: +1-212-998-9638 (S.R.); +1-212-998-9597 (T.G.B.)

Academic Editors: Zikri Arslan and Michael Bolshov
Received: 16 August 2020; Accepted: 19 October 2020; Published: date

Table S1. Freshwater (FW) sample codes: associating with rivers and villages of Upolu Island, Samoa

Sample code	River/Lake	Nearest Village/River Level	Brackish Samples	Sample code	River/Lake	Nearest Village/River Level	Brackish Samples
FW 01	Afuilo Dam South	Headwaters		FW 35	Leuso	Laulii	
FW 02	Eva	Upstream		FW 36	Leuso	Laulii	
FW 03	Eva	Eva		FW 37	Lona	Lona	
FW 04	Fagalii	Headwaters		FW 38	Lona	Lona	
FW 05	Fagalii	Fagali'i-uta		FW 39	Nu'usuatia	Headwaters	
FW 06	Fagalii	Fagali'l	**Mangrove**	FW 40	Mulivaifagatola	Headwaters	
FW 07	Faleaseela	Headwaters		FW 41	Mulivaifagatola	Upstream	
FW 08	Faleaseela	Upstream		FW 42	Mulivaifagatola	Salani	**Estuary**
FW 09	Faleaseela	Faleaseela		FW 43	Namo	Solosolo	
FW 10	Falefa	Falevao		FW 44	Namo	Solosolo	
FW 11	Falefa	Falefa		FW 45	Namo	Solosolo	
FW 12	Falefa	Falefa		FW 46	Piu	Upstream	
FW 13	Falefa	Falefa	**Estuary**	FW 47	Piu	Malaemalu	
FW 14	Fuluasou	Alafua		FW 48	Solo	Upstream	
FW 15	Fuluasou	Tuanaimato		FW 49	Solo	Solosolo	
FW 16	Fuluasou	Lepea		FW 50	Taelefaga	Taelefaga	
FW 17	Gasegase	Moamoa		FW 51	Tafitoala	Headwaters	
FW 18	Gasegase	Moamoa		FW 52	Tafitoala	Upstream	
FW 19	Gasegase	Fugalei		FW 53	Tiavea	Headwaters	
FW 20	Laulii	Vailele		FW 54	Tiavea	Tiavea	
FW 21	Laulii	Vailele		FW 55	Tiavea	Downstream	
FW 22	Laulii	Vailele		FW 56	Tiavea	Downstream	
FW 23	Laulii	Laulii		FW 57	Togitogiga	Headwaters	
FW 24	Lotofaga	Upstream		FW 58	Togitogiga	Upstream	
FW 25	Lotofaga	Lotofaga		FW 59	Togitogiga	Saleilua	
FW 26	Lotofaga	Satao	**Estuary**	FW 60	Vailima	Vaola	
FW 27	Lepa	Upstream		FW 61	Vailima	Malifa	
FW 28	Lepa	Aufaga		FW 62	Vailima	Mulivai	
FW 29	Letogo	Headwaters		FW 63	Vailima	Mulivai	
FW 30	Letogo	Upstream		FW 64	Vaisigano	Upstream	
FW 31	Letogo	Upstream		FW 65	Vaisigano	Malifa, Lelata	
FW 32	Letogo	Letogo		FW 66	Lake Lanoanea	Headwaters	
FW 33	Letogo	Letogo	**Mangrove**	FW 67	Lake Lanutoo	Headwaters	
FW 34	Letogo	Letogo	**Mangrove**				

Table S2. Mangrove Swamp water (MW) sample codes: associating with rivers and villages of Upolu Island, Samoa

Sample code	Nearest River	Nearest Village	Brackish Samples
FW 06[b]	Fagalii	Fagali'l	**Mangrove**
MW 01[a]	Faleaseela	Samatau	**Mangrove**
FW 13[b]	Falefa	Falefa	**Estuary**
MW 02[a]	Fuluasou	Salemoa	**Mangrove**
FW 26[b]	Lotofaga	Satao	**Estuary**
FW 33[b]	Letogo	Letogo	**Mangrove**
FW 34[b]	Letogo	Letogo	**Mangrove**
MW 03[a]	Mulivaifagatola	Matatufu	**Mangrove**
FW 42[b]	Mulivaifagatola	Salani	**Estuary**

[a]These MW samples are from shoreline mangrove areas, and are not included in any FW or SW sample list. The other 6 samples are also listed in FW list too.

[b]These FW samples are mangrove swamps associated with a river or are samples situated within an estuary. These samples are included in the FW sample list as well.

Table S3. Saltwater (SW) sample codes: associating with rivers and villages of Upolu Island, Samoa

Sample Code	Nearest River	Nearest Village	Sample Code	Nearest River	Nearest Village	Sample Code	Nearest River	Nearest Village
SW 001	Eva	Eva	SW 037	Lepa	Lepa	SW 073	Valima	Apia
SW 002	Eva	Eva	SW 038	Letogo	Letogo	SW 074	Valima	Apia
SW 003	Eva	Salelesi	SW 039	Letogo	Letogo	SW 075		Apolima
SW 004	Eva	Salelesi	SW 040	Leuso	Leusoalii	SW 076		Apolima
SW 005	Fagalii	Fagalii	SW 041	Leuso	Leusoalii	SW 077		Apolima
SW 006	Fagalii	Fagalii	SW 042	Leuso	Luatuanuu	SW 087		Lalomanu
SW 007	Faleaseela	Faleaseela	SW 043	Leuso	Luatuanuu	SW 079		Lalomanu
SW 008	Faleaseela	Faleaseela	SW 044	Leuso	Luatuanuu	SW 080		Lalomanu
SW 009	Faleaseela	Faleletai	SW 045	Lona	Uafato	SW 081		Malaela
SW 010	Faleaseela	Faleletai	SW 046	Lona	Uafato	SW 082		Malaela
SW 011	Faleaseela	Faleletai	SW 047	Mulivaifagatola	Satalo	SW 083		Matatufu
SW 012	Falefa	Sauago/Saletele	SW 048	Mulivaifagatola	Satalo	SW 084		Matatufu
SW 013	Falefa	Sauago/Saletele	SW 049	Mulivaifagatola	Satalo	SW 085		Matatufu
SW 014	Falefa	Sauago/Saletele	SW 050	Piu	Malaemalu	SW 086		Mulifanua
SW 015	Falefa	Falefa	SW 051	Solo	Solosolo	SW 087		Mulifanua
SW 016	Falefa	Falefa	SW 052	Solo	Solosolo	SW 088		Salani
SW 017	Fuluasou	Afega	SW 053	Taelafaga	Taelafaga	SW 089		Salani
SW 018	Fuluasou	Afega	SW 054	Taelafaga	Taelafaga	SW 090		Samatao
SW 019	Fuluasou	Faleasiu	SW 055	Taelafaga	Taelafaga	SW 091		Samatao
SW 020	Fuluasou	Faleasiu	SW 056	Tafitoala	Tafitoala	SW 092		Samatao
SW 021	Fuluasou	Faleasiu	SW 057	Tafitoala	Tafitoala	SW 093		Samatao
SW 022	Fuluasou	Faleula	SW 058	Tafitoala	Tafitoala	SW 094		Siumu
SW 023	Fuluasou	Faleula	SW 059	Tafitoala	Tafitoala	SW 095		Siumu
SW 024	Fuluasou	Faleula	SW 060	Tiavia	Tiavia	SW 096		Siumu
SW 025	Fuluasou	Faleula	SW 061	Tiavia	Tiavia	SW 097		Vavau
SW 026	Fuluasou	Faleula	SW 062	Tiavia	Tiavia	SW 098		Vavau
SW 027	Fuluasou	Faleula	SW 063	Vaisigano	Apia	SW 099		Vavau
SW 028	Fuluasou	Saleimoa	SW 064	Vaisigano	Apia	SW 100		
SW 029	Fuluasou	Saleimoa	SW 065	Vaisigano	Apia	SW 101		
SW 030	Laulii	Vailele	SW 066	Valima	Apia	SW 102		
SW 031	Lotofaga	Salamumu	SW 067	Valima	Apia	SW 103		
SW 032	Lotofaga	Salamumu	SW 068	Valima	Apia	SW 104		
SW 033	Lotofaga	Salamumu	SW 069	Valima	Apia	SW 105		
SW 034	Lotofaga	Satao	SW 070	Valima	Apia	SW 106		
SW 035	Lotofaga	Satao	SW 071	Valima	Apia			
SW 036	Lepa	Lepa	SW 072	Valima	Apia			

Table S4. Mitigation ranking by village and river. Based on freshwater and saltwater element concentrations, which relate to harmful chemicals, and on elevated salinity levels reported in this study, we subjectively ranked the urgency for mitigation and color coded accordingly the river names in Figure 4 as illustrated in this scheme

Village	River	Rank Order	Village	River	Rank Order
Apia	Vailima	**Mitigation Need Extreme**	Apolima	Lona	**Mitigation Need Indicated**
Falefa	Falefa	**Mitigation Need Urgent**	Lona	Gasegase	
Vavau			Moamoa		
Satalo	Mulivaifagatola		Nu'utele island	Faleaseela	
Lalomanu			Samatau	Lepa	
Samatau			Aufaga	Fagalii	
Letogo	Letogo		Fagali'i-uta	Gasegase	
Faleula	Fuluasou		Fugalei	Fuluasou	
Taelafaga	Taelafaga		Lepea	Leuso	
Tafitoala	Tafitoala	**Mitigation Need Strong**	Luatuanuu	Eva	
Apia	Vaisigano		Salelesi		
Fagalii	Fagalii		Sinuleie	Fuluasou	
Faleaseela	Faleaseela		Tuanaimato	Vailima	
Malaemalu	Piu		Vaola	Fuluasou	
Afega	Fuluasou		Alafua	Fuluasou	
Eva	Eva		Faleasiu	Lepa	
Faleletai	Faleaseela		Lepa	Leafe	
Malifa	Vailima		Lotofaga		
Matatufu	Mulivaifagatola		Mulifanua	Leafe	
Mulivai	Vailima		Sataoa	Namo	
Salamumu	Leafe		Solosolo	Lona	
Salani	Mulivaifagatola		Uafato	Falefa	
Saleimoa	Fuluasou		Falevao	Laulii	
Siumu			Laulii	Togitogiga	
Solosolo	Solo		Saleilua	Vaisigano	
Tiavea	Tiavea		Malifa, Lelata		
Vailele	Laulii		Malaela		
Downstream	Tiavea		Sauago/Saletele	Falefa	
Leusoalii	Leuso				

Table S5. Limit of detection, correlation coefficient of calibration plots for each elements, and selected isotopes for the determination of elemental concentration in various water samples of Upolu Island, Samoa

Element	LoD[a] (μg/L)	Corr. Coaf.[b]	Selected isotope	Element	LoD[a] (μg/L)	Corr. Coaf.[b]	Selected isotope
Li	0.14	0.9999	7 Li	Cd	0.038	0.9999	111 Cd
Be	1.8	1	9 Be	In	0.0017	0.9999	115 In
B	0.27	0.9997	10 B	Sn	0.016	1	118 Sn
Na	0.26	0.9999	23 Na	Sb	0.013	1	121 Sb
Mg	0.27	0.9998	24 Mg	Te	0.108	0.9999	128 Te
Al	0.029	1	27 Al	Cs	0.0037	0.9999	133 Cs
Si	1.1	0.9994	28 Si	Ba	0.0032	0.9999	138 Ba
P	3.17	1	31 P	La	0.0092	0.9998	139 La
S	207	0.9988	34 S	Ce	0.0022	0.9998	140 Ce
Cl	63.6	1	35 Cl	Pr	0.0027	0.9998	141 Pr
K	1.2	0.9999	39 K	Nd	0.011	0.9998	143 Nd
Ca	2.5	0.9994	44 Ca	Sm	0.010	0.9998	149 Sm
Ti	0.36	0.9999	49 Ti	Eu	0.0030	0.9998	151 Eu
V	0.021	0.9996	51 V	Gd	0.016	0.9997	157 Gd
Cr	0.0074	0.9995	52 Cr	Tb	0.0025	0.9998	159 Tb
Fe	0.095	0.9999	54 Fe	Dy	0.0087	0.9997	163 Dy
Mn	0.011	0.9997	55 Mn	Ho	0.0012	0.9997	165 Ho
Ni	0.021	0.9999	58 Ni	Er	0.0021	0.9997	166 Er
Co	0.016	0.9996	59 Co	Tm	0.0025	0.9997	169 Tm
Cu	0.044	1	65 Cu	Yb	0.0016	0.9997	173 Yb
Zn	0.086	0.9998	66 Zn	Lu	0.0014	0.9997	175 Lu
Ga	0.023	0.9999	71 Ga	Hf	0.0069	0.9998	178 Hf
As	0.045	0.9999	75 As	Ta	0.00084	0.9999	181 Ta
Br	0.69	0.9996	79 Br	W	0.011	0.9999	182 W
Se	0.49	0.9999	82 Se	Re	0.0033	0.9999	185 Re
Rb	0.0054	0.9999	85 Rb	Os	0.011	0.9999	189 Os
Sr	0.0017	0.9999	88 Sr	Ir	0.0017	0.9999	193 Ir
Y	0.0057	0.9998	89 Y	Pt	0.011	0.9999	195 Pt
Zr	0.0077	0.9999	90 Zr	Au	0.021	0.9999	197 Au
Nb	0.0051	0.9999	93 Nb	Hg	0.020	0.9982	202 Hg
Mo	0.030	0.9999	95 Mo	Tl	0.0019	0.9999	205 Tl
Ru	0.011	1	101 Ru	Pb	0.0064	0.9999	208 Pb
Ag	0.0067	0.9999	107 Ag	Bi	0.0069	0.9999	209 Bi
Pd	0.012	1	108 Pd	U	0.0045	0.9999	238 U

[a]LoD: Limit of Detection; [b]Corr. Coef.: Correlation coefficient of calibration plots (R^2).

Table S6. Certified and obtained values for the standard reference material (SRM) of NIST 1640a Trace Elements in Natural Water

Element	Certified value[a] ± SD[b]	Obtained value[a] ± SD[c]	Recovery[d] (%)
Be	3.026 ± 0.028	2.8 ± 0.061	93
B	303.1 ± 3.1	303 ± 0.61	100
Al	53.0 ± 1.8	52 ± 0.26	98
V	15.05 ± 0.25	15 ± 0.021	102
Cr	40.54 ± 0.30	42 ± 0.044	104
Fe	36.8 ± 1.8	34 ± 0.14	93
Mn	40.39 ± 0.36	38 ± 0.23	95
Co	20.24 ± 0.24	21 ± 0.024	106
Ni	25.32 ± 0.14	25 ± 0.080	99
Cu	85.75 ± 0.51	87 ± 0.092	101
Zn	55.64 ± 0.35	58 ± 0.18	104
As	8.075 ± 0.070	8.5 ± 0.05	105
Se	20.13 ± 0.17	22 ± 0.36	108
Sr	126.03 ± 0.91	119 ± 0.25	94
Mo	45.60 ± 0.61	45 ± 0.018	99
Ag	8.081 ± 0.046	7.9 ± 0.013	98
Cd	3.992 ± 0.074	3.8 ± 0.016	95
Sb	5.105 ± 0.046	5.2 ± 0.018	102
Ba	151.80 ± 0.83	149 ± 0.42	98
Tl	1.619 ± 0.016	1.5 ± 0.0040	92
Pb	12.101 ± 0.050	12 ± 0.047	98
U	25.35 ± 0.27	24 ± 0.057	95

[a]values: all concentration values are in µg/L; [b]SD: standard deviation; [c]SD: standard deviation (n=3); [d]Recovery calculated based on: obtained value/certified value*100.

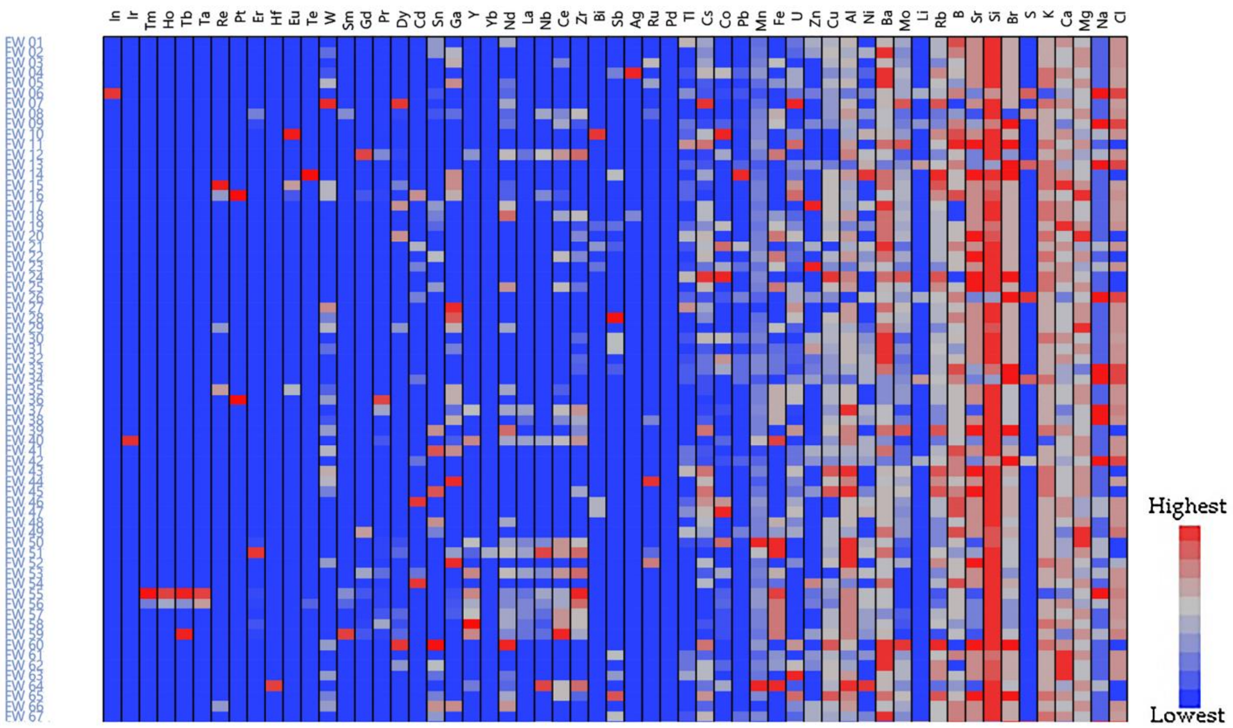

Figure S1. Log transformed and quantile normalized element concentration data in freshwater samples. Trace concentrations range between dark blue (not detected) and dark red (maximum concentration). The legend to the right shows the color scale of the data representing arbitrary (normalized) values from low (blue) to high (red) and not absolute element concentrations. The element symbol is above its own colored column. Each row in the plot is an individually collected freshwater (FW) sample, and given in the left margin of each row is its sample number for associating with rivers and villages of Upolu Island (see Supplementary Table S1).

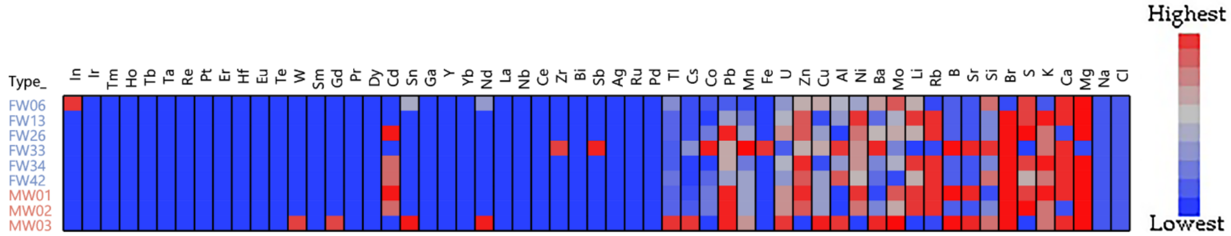

Figure S2. Log transformed and quantile normalized element concentration data in mangrove swamp water samples. Trace concentrations range between dark blue (not detected) and dark red (maximum concentration). The legend to the right shows the color scale of the data representing arbitrary (normalized) values from low (blue) to high (red) and not absolute element concentrations. The element symbol is above its own colored column. Each row in the plot is an individually collected mangrove swamp water (MW) sample, and given in the left margin of each row is its sample number for associating with rivers and villages of Upolu Island (see Supplementary Table S2).

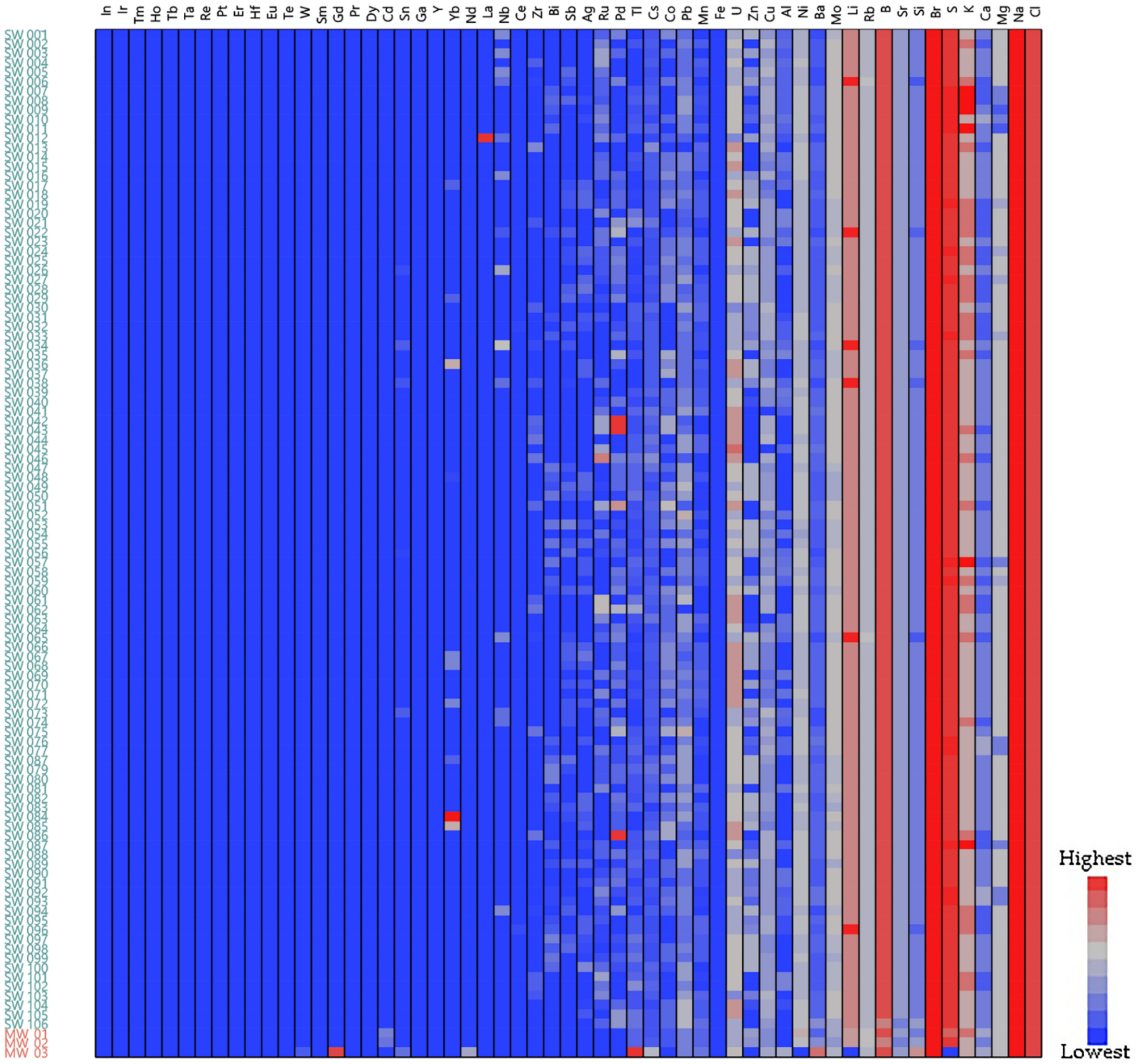

Figure S3. Log transformed and quantile normalized element concentration data in 106 saltwater samples and 3 MW samples. Trace concentrations range between dark blue (not detected) and dark red (maximum concentration). The legend to the right shows the color scale of the data representing arbitrary (normalized) values from low (blue) to high (red) and not absolute element concentrations. The element symbol is above its own colored column. Each row in the plot is an individually collected saltwater (SW) sample, and given in the left margin of each row is its sample number for associating with rivers and villages of Upolu Island (see Supplementary Table S3).
